# Case Report: A novel desmoplakin mutation in a taiwanese woman with familial dilated cardiomyopathy that necessitated heart transplantation

**DOI:** 10.3389/fgene.2022.954931

**Published:** 2022-09-23

**Authors:** Yi-Han Chang, Pei Lin, Jia-Ling Lin, Hsin-Yu Huang, Chao-Kai Hsu, Chih-Hsin Hsu

**Affiliations:** ^1^ Department of Dermatology, National Cheng Kung University Hospital, College of Medicine, National Cheng Kung University, Tainan, Taiwan; ^2^ Education Center, National Cheng Kung University Hospital, College of Medicine, National Cheng Kung University, Tainan, Taiwan; ^3^ International Research Center of Wound Repair and Regeneration (iWRR), National Cheng Kung University, Tainan, Taiwan; ^4^ Division of Cardiology, Department of Internal Medicine, Tainan Municipal An-Nan Hospital, China Medical University, Tainan, Taiwan; ^5^ Division of Cardiology, Department of Internal Medicine, National Cheng Kung University Hospital Dou-Liou Branch, College of Medicine, National Cheng Kung University, Tainan, Taiwan; ^6^ Division of Cardiology, Department of Internal Medicine, National Cheng Kung University Hospital, Tainan, Taiwan; ^7^ Institute of Clinical Medicine, College of Medicine, National Cheng Kung University, Tainan, Taiwan; ^8^ Division of Critical Care, Department of Internal Medicine, National Cheng Kung University Hospital, College of Medicine, National Cheng Kung University, Tainan, Taiwan

**Keywords:** familial dilated cardiomyopathy (FDC), desmoplakin gene (DSP), heart transplantation (HTx), whole exome sequencing, dilated cardiomyopathy (DCM)

## Abstract

Around one-third of patients diagnosed with idiopathic dilated cardiomyopathy (DCM) turn out to be familial cases, in only a few of which the identification of a pathogenic/likely pathogenic variant could be achieved. Cardiomyopathy caused by desmoplakin gene mutations represents a distinct form with a high prevalence of left ventricle involvement. We report a novel desmoplakin mutation carried by two individuals in a Taiwanese family, in which the proband recovered well after heart transplantation and under medical control, while her son had received an implantable cardioverter defibrillator and has been under guideline-directed medical therapy. The present study broadens the genetic spectrum of this disease entity and strengthens the notion that a detailed family history with genetic study contributes to the early detection and treatment of inherited diseases.

## Introduction

Dilated cardiomyopathy (DCM) is one of the most common causes of heart failure and is a leading diagnosis among heart transplant recipients (Cuenca et al., 2016). DCM is characterized by left ventricular or biventricular dilation and impaired systolic function that is not explained by abnormal loading conditions (e.g., hypertension or valvular heart disease) nor by coronary artery disease (Schultheiss et al., 2019). The underlying etiologies of DCM include genetic mutations, infection, autoimmune disease, toxin exposure, metabolic or endocrine dysfunction, neuromuscular disease, and pregnancy/peripartum cardiomyopathy. In some cases, however, there is no recognizable cause even after thorough assessments. These cases are given the clinical diagnosis of “idiopathic” DCM. Previous studies have demonstrated that familial DCM accounts for 20%–35% of patients who are initially classified as idiopathic (Petretta et al., 2011; Hershberger et al., 2013). Therefore, the guidelines of the Heart Failure Society of America/American College of Medical Genetics and Genomics recommend genetic testing for all patients with idiopathic DCM, regardless of family history (Hershberger et al., 2018).

Desmoplakin (DSP) is a cytoskeletal linker protein member of the plakin family. Its main function is to connect intermediate filament proteins with other desmosomal components, playing an important role in cell–cell adhesion. Structurally intact desmosomes provide mechanical strength and resilience, which are crucial to combat stress in the epidermis and the heart (Garrod & Chidgey, 2008). According to previous studies, *DSP* (6p24.3) mutations are mostly associated with arrhythmogenic right ventricular cardiomyopathy (ARVC; OMIM #607450), which primarily affects the structure and function of right ventricle (RV), with clinical manifestations of right and/or left ventricular ectopy and sudden cardiac death, and with pathological features of fibro-fatty replacement (Rampazzo et al., 2002; Azaouagh et al., 2011). Recent studies suggest that DSP cardiomyopathy represents an entity distinct from typical forms of ARVC or DCM. Instead, DSP cardiomyopathy is characterized by early and predominant left ventricle (LV) involvement, frequent ventricular arrhythmia, and a specific fibrosis pattern seen in magnetic resonance imaging (Castelletti et al., 2017; Helio et al., 2020; Smith et al., 2020).

In the present study, we report a female patient with familial DCM who underwent heart transplantation. Genetic study identified a novel *DSP* mutation, c.6384delG, p.(Glu2128SerfsTer18), in the proband and her son. Consistent with the current concept of DSP cardiomyopathy, these two cases exhibited impairments in LV structure and function.

## Methods

### Whole-exome sequencing

Germline DNA extracted from the proband and her family members (the proband’s two sons and her younger sister) was used for paired-end library preparation using the SureSelect All Exon 50 Mb Version 4.0 kit (Agilent, Santa Clara, CA, United States) according to the manufacturer’s recommendations. Sequencing as carried out by massively parallel sequencing with 100-bp paired-end reads using the HiSeq-2000 platform (Illumina, CA, United States). The Novoalign software package (Novocraft Technologies Sdn Bhd) was used to align reads generated to the reference human genome. Reads mapping to multiple locations on the reference human genome were excluded from downstream analysis. The BedTools package was used to calculate the depth and breadth of sequence coverage (Quinlan & Hall, 2010). Single-nucleotide substitutions and small indels were detected with the SamTools package (Li et al., 2009). Sequence variants were annotated with the Annovar tool (Wang et al., 2010). To assess the pathogenicity of the candidate variants, an in-house variant-filtering pipeline was used. Nonsense variants or indels resulting in frameshift mutations with minor allele frequencies (MAF) of less than 0.5% in the 1,000 Genomes Project (Siva, 2008) and Exome Aggregation Consortium (ExAC) were included. The damage prediction criteria for filtering the candidate variants included a Combined Annotation Dependent Depletion (CADD) score of above 15, a Deleterious Annotation of Genetic variants using Neural Networks (DANN) score of above 0.95, and a Polymorphism Phenotyping v2 (PolyPhen-2) score of above 0.95. Variants with MAF exceeding 0.5% or with damage prediction scores not fulfilling our criteria were excluded as non-pathogenic. BAM files of WES were visualized *via* Integrative Genomics Viewer (IGV) (Robinson et al., 2011).

### Sanger sequencing

Confirmative polymerase chain reaction (PCR) and Sanger sequencing tests were performed on the DNA from the proband and her family members (the proband’s two sons and her younger sister) to validate the filtered variants detected by WES and for segregation analysis. Primers were designed using the Ensembl database (Howe et al., 2021) and Primer3 (Untergasser et al., 2012) online software.

## Results

**FIGURE 1:**
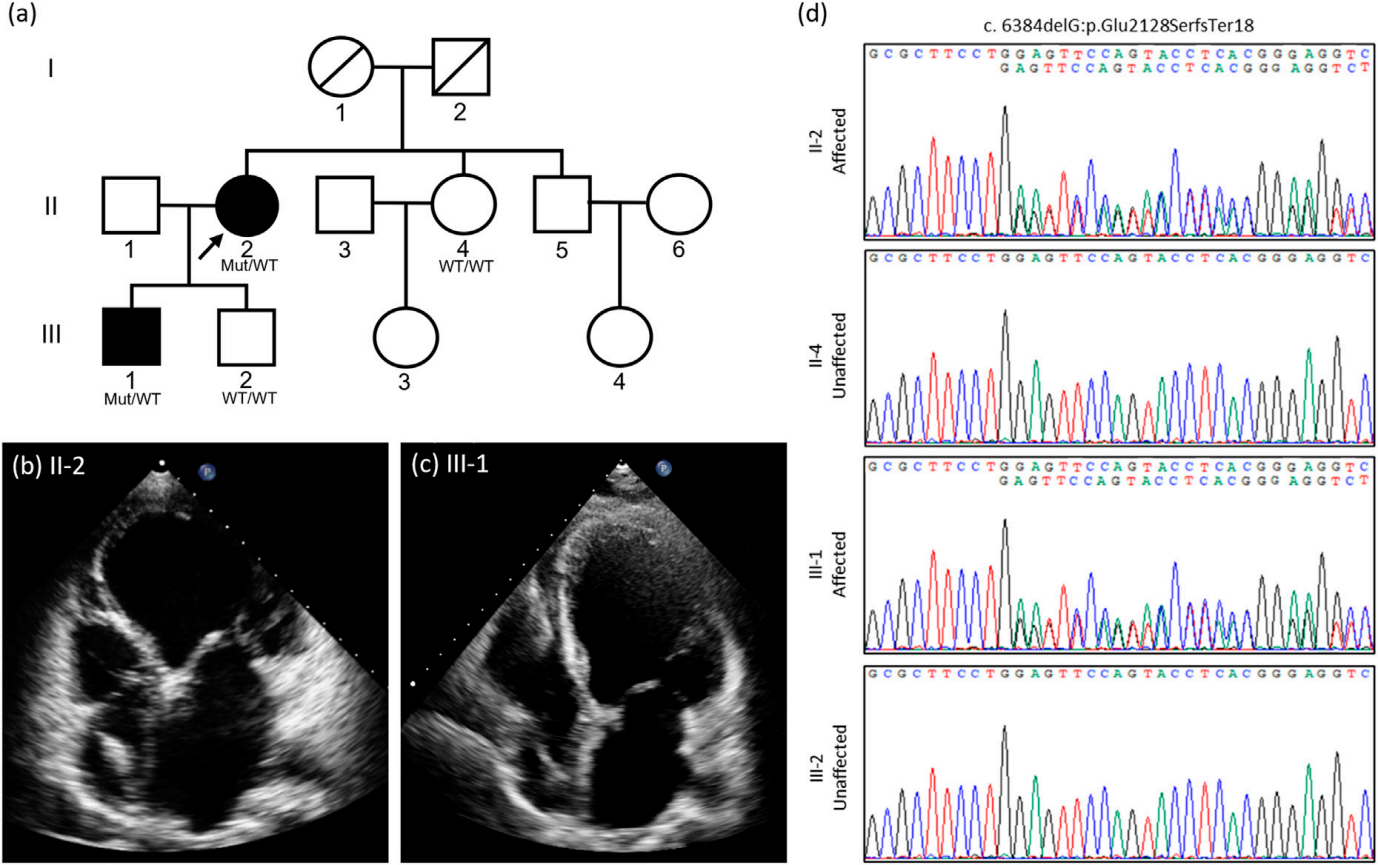
The pedigree and cardiac examinations of the proband (II-2) and her son (III-1). **(A)** The pedigree of the proband (arrow) and her family. **(B)** Echocardiography of the proband (II-2) shows dilated LV and LA with LVEDD 78 mm and poor LV function, with EF 32% and ICD lead *in situ*. **(C)** Echocardiography of the proband’s son (III-1) shows dilated LV and LA with LVEDD 78 mm and poor LV function with EF 22.8%, with the ICD lead *in situ*. **(D)** Genetic study of the proband’s family. Sanger sequencing reveals a deletion mutation in the proband (II-2) and her son (III-1), suggesting a dominant inheritance pattern for this variant. EF: ejection fraction; ICD: implantable cardioverter defibrillator; LA: left atrium; LV: left ventricle; LVEDD: left ventricular end-diastolic diameter.

A 49-year-old female presented to our hospital with dyspnea on exertion and chest tightness for 2 months (Figure 1A). Physical examination found a 2/6 pansystolic murmur over the left lower sternal border and apex. Electrocardiography showed sinus rhythm with left anterior hemiblock and left ventricular hypertrophy. Chest X-ray showed cardiomegaly. In echocardiography, her left ventricular end-diastolic diameter (LVEDD) was 78 mm and the ejection fraction by Simpson’s biplane method was 32% with moderate mitral regurgitation (Figure 1B). Coronary angiography showed normal coronary arteries. Radionuclide ventriculography showed impaired systolic and diastolic function of the LV with global ejection fraction 25% but normal systolic function of the RV. She was diagnosed with DCM. Due to frequent ventricular extrasystoles and non-sustained ventricular tachycardia, she received an implantable cardioverter defibrillator. She was treated with guideline-directed medical therapy for heart failure, including a beta blocker, an angiotensin-converting enzyme inhibitor (ACEi), a mineralocorticoid receptor antagonist, and ivabradine. Later, the ACEi was replaced with an angiotensin receptor–neprilysin inhibitor. Her heart function and daily performance did not improve, but deteriorated rapidly in the following 2 years. The highest N-terminal pro-brain natriuretic peptide (NT-pro-BNP) concentration was 7,000 ng/L. At the age of 59 years, she received a heart transplant. Currently, she is in healthy condition under immunosuppressants with mycophenolate, tacrolimus, and prednisolone.

Notably, the proband’s son was diagnosed with DCM at the age of 24 years with ejection fraction 22.8% and LVEDD 78.2 mm in the initial echocardiography (Figure 1C). Radionuclide ventriculography also showed impaired systolic and diastolic function of the LV with global ejection fraction 26% but normal systolic function of the RV. He also presented with frequent ventricular extrasystoles and non-sustained ventricular tachycardia and received an implantable cardioverter defibrillator. He has been under guideline-directed medical therapy for 10 years. He was in New York Heart Association classification II, and his ejection fraction did not improve much. The highest level of NT-pro-BNP was 600 ng/L.

Familial DCM was highly suspected, considering the family history. Therefore, molecular genetic studies were arranged and a novel mutation in the *DSP* gene was identified. With informed consent, we collected peripheral blood from the two patients and other family members (the proband’s second son and her younger sister), and genomic DNA was extracted for further mutation analysis. Whole-exome sequencing initially identified three potential variant candidates (laminin alpha-4, aminoacyl-tRNA synthetase 2, and *DSP*), and subsequent segregation analysis confirmed the *DSP* variant to be the culprit, with an autosomal dominant inheritance pattern. This unreported heterozygous deletion, c.6384delG (p.Glu2128SerfsTer18), in *DSP* (NM_001008844) leads to premature termination codon (PTC) formation (Figure 1D). The diagnosis of desmoplakin cardiomyopathy in these two patients was confirmed based on the clinical manifestations of LV systolic dysfunction, the electrocardiographic findings of frequent ventricular extrasystoles and non-sustained ventricular tachycardia, and the positive results of genetic analysis, with a novel heterozygous deletion mutation in *DSP* in this family.

## Discussion

**FIGURE 2:**
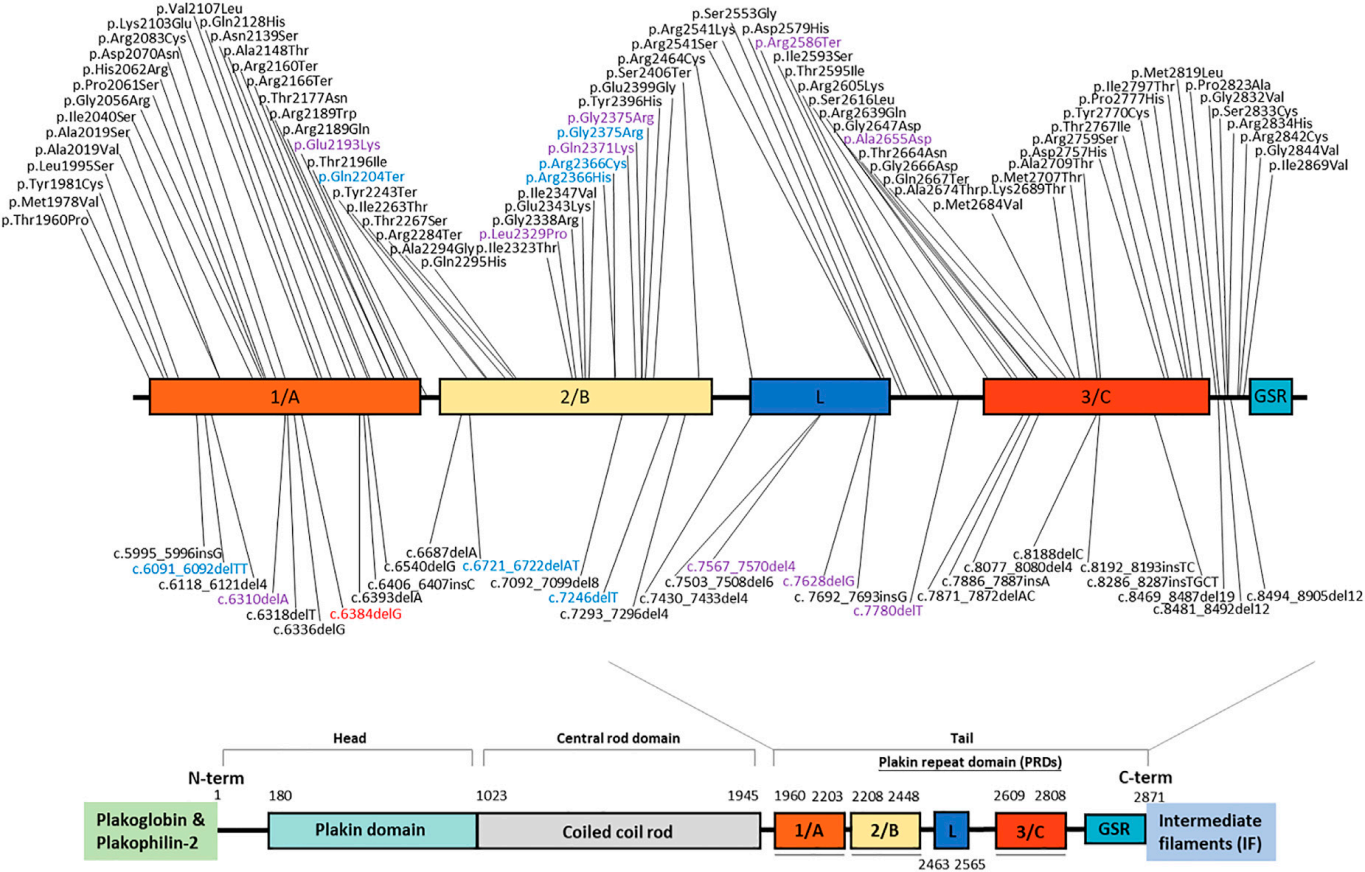
Schematic of desmoplakin protein domains labeled with reported mutations within the tail domain. The mutation identified in our study is marked in red. Mutations with different colors account for different phenotypes (black: heart only; blue: skin only; purple: heart and skin). Variants above the schematic are missense or nonsense mutations; those below the schematic are indel mutations.

Based on the Human Gene Mutation Database, more than 300 *DSP* mutations had been documented as of October 2020. These include nonsense/missense, insertion, deletion, and regulatory mutations, with diverse phenotypes (Figure 2). The unreported mutation identified in our study is a deletion mutation that is situated within the plakin repeat domain (PRD 1/A) of the tail domain. Consequential PTC formation leads to truncated desmoplakin protein or induces nonsense-mediated decay. Since that the C-terminus of desmoplakin is responsible for interactions with intermediate filaments, it is plausible that this mutation changes the structure of the carboxyl domain and further hinders anchorage between desmoplakin and intermediate filaments.

Mutations in human *DSP* can present as abnormalities in the skin, hair, and heart, emphasizing the importance of desmoplakin against continuous mechanical stress (Garrod & Chidgey, 2008). Of note, no skin or hair abnormalities were noted in our two cases, and no such defects were mentioned in other family members. This newly identified mutation seems to manifest a heart-only phenotype, based on the clinical history. To confirm the phenotypic landscape, a longer follow-up period for this family or observation from any other cases with this mutation is needed. The mutation itself did not add more information on the clinical phenotype, because previous studies (Mahoney et al., 2010; Pigors et al., 2015) demonstrated that individual *DSP* mutations are scattered throughout the coding sequence and that there is no clear association between mutation location, type, and clinical presentation (Figure 2).

In the present study, familial disease was suspected because the proband’s son was diagnosed with DCM at age 24. We took a thorough clinical history of the family and performed genetic analyses. A novel pathogenic *DSP* mutation with autosomal dominant inheritance was identified, with the clinical presentation of LV dysfunction and ventricular ectopy that are typical of desmoplakin cardiomyopathy (Smith et al., 2020). The findings are consistent with the current concept that *DSP* mutations seem to cause a unique form of cardiomyopathy with a high prevalence of LV involvement (Norman et al., 2005; Bhonsale et al., 2015; Pigors et al., 2015; Helio et al., 2020), especially if the patient is a carrier of a *DSP* nonsense mutation (Sen-Chowdhry et al., 2007; Lopez-Ayala et al., 2014; Castelletti et al., 2017).

DCM is the most frequent cause of heart transplantation, with approximately 20%–25% of transplant recipients having direct evidence of familial disease (Cuenca et al., 2016). A retrospective study using a large, contemporary, nationwide database that aimed to investigate the outcome of familial DCM patients after heart transplantation (Khayata et al., 2019) demonstrated that these patients have a higher risk of early rejection but are more likely to survive than are patients with ischemic cardiomyopathy (ICM). These findings could be explained by less hepatic or renal dysfunction in familial DCM patients and more comorbidities in ICM patients (Grimm et al., 2015; Shore et al., 2015).

Certain types of DCM have unique presentations. For example, patients with *LMNA* mutations possess higher rates of conduction block, ventricular arrhythmia, and sudden cardiac death (Schultheiss et al., 2019). Genetic study helps physicians to define the clinical course of this complicated disease and to identify patients who need more tailored therapies. Also, patients with DCM may have a clinically indolent phase during the early stage of the disease (Kinnamon et al., 2017). With the advent of next-generation sequencing technology (Yeh, 2019), we are able to provide better genetic counseling for patients and their families. Apart from confirming both the clinical diagnosis and the inheritance pattern, genetic study is important in terms of evaluating the risk for future offspring as well as achieving the goal of early diagnosis by enabling early optimal treatment to delay the disease progression and to prevent severe complications (McMurray, 2015; Fatkin et al., 2017; Kinnamon et al., 2017; Rivero et al., 2017).

In conclusion, we reported the case of a Taiwanese woman with familial DCM and a novel *DSP* mutation. Our findings broaden the genetic spectrum of this disease entity and strengthen the notion that a detailed family history, including genetic study, can contribute to the early detection and treatment of inherited diseases.

## Data Availability

The datasets for this article are not publicly available due to concerns regarding participant/patient anonymity. Requests to access the datasets should be directed to the corresponding author.
